# Neonatal maple syrup urine disease case report and literature review

**DOI:** 10.1097/MD.0000000000032174

**Published:** 2022-12-16

**Authors:** Qiao Liu, Fang Li, Jingjing Zhou, Xiaoyu Liu, Jidong Peng, Lianggeng Gong

**Affiliations:** a Department of Medical Imaging Center, Second Affiliated Hospital of Nanchang University, Nanchang, China; b Department of Medical Imaging Center, Ganzhou People’s Hospital, Ganzhou, China.

**Keywords:** diffusion-weighted imaging, genetic metabolic disease, hypoglycemic encephalopathy, magnetic resonance imaging, maple syrup urine disease

## Abstract

The main clinical symptoms of maple syrup urine disease (MSUD) are dehydration, acidosis, nervous system symptoms and intellectual disability. The brain imaging findings were mainly caused by cytotoxic edema. The lesions usually occur at the site consistent with the myelination process of normal neonates. The distribution is mostly symmetric, and the diffusion is obviously limited.

**Patient concerns:**

Herein, we report a rare case of an 8-day-old female patient who presented with abnormal symptoms, such as difficulty eating, convulsions, slow reaction, difficulty in correcting hypoglycemia and severe metabolic disorders. Brain magnetic resonance imaging (MRI) revealed abnormal signal intensity mainly involving the brainstem, cervical spinal cord, bilateral cerebellar hemispheres, basal ganglia, thalamus, precentral gyrus, and postcentral gyrus with characteristic hyperintensity on diffusion-weighted imaging (DWI) sequence. MSUD is rarely reported, while cervical spinal cord involvement is extremely rare.

**Diagnoses:**

Blood tandem mass spectrometry, urine organic acid detection, and genetic disease overall genetic tests were performed to further confirm the diagnosis of MSUD.

**Interventions:**

Under general anesthesia, she underwent open surgical procedures for liver transplantation.

**Outcomes:**

The child was in a stable condition after liver transplantation, and the diet was not restricted.

**Lessons:**

MSUD in neonates is rare. Our case report and literature review was aim to describe the clinic and imaging characteristics of it, and highlight physicians must be aware of this entity in newborns so as to reduce misdiagnosis due to unfamiliarity.

## 1. Introduction

Maple syrup urine disease (MSUD) was first reported by pediatrician Menkes in 1954, as the α-ketoacid excreted in urine smells like maple syrup. MSUD is a rare genetic disorder which manifested as impaired branched-chain amino acid (BCAA) metabolism caused by branched-chain α-ketoacid dehydrogenase (BCKD) complex deficiency.^[1]^ The BCKD complex is thought to be composed of a core of 24 transacylase (E2) subunits, and associated decarboxylase (E1), dehydrogenase (E3), and regulatory subunits. Dihydrolipoamide branched chain transacylase E2(DBT) encodes the transacylase (E2) subunit. Three MSUD genotypes have been identified to date based on the genes involved: subtype 1α mutation affecting the Eα (BCKDHA) gene, subtype 1β mutation Eβ (BCKDHB) and subtype II mutation E2 (DBT) gene.^[2]^ Metabolic acidosis is the main cause of death in the first year while survivors often suffer from mental retardation, spastic palsy, cortical blindness and other neurological disorders. MSUD can be divided into five types according to the central brain injury and metabolic acidosis: classic type, intermediate type, intermittent type, thiamin (vitamin B1, VB1) active type and dihydrothioctinamide acyl dehydrogenase (E3) type. The last four types are also called delayed type.^[3,4]^ Most adolescents and young adults with MSUD have different severity of magnetic resonance imaging (MRI) white matter changes.^[5]^ The abnormal MRI findings were mainly increased signal intensity in T2-weighted images of the midbrain, brainstem, thalamus and globus pallidus. This myelin dysplasia is a secondary change of long-term exposure to BCAA.^[5,6]^ The mechanism of brain damage in MSUD patients is still unclear. Some scholars believe that the accumulation of BCAA in the brain will inhibit the activity of pyruvate dehydrogenase and α-ketoglutarate dehydrogenase which destroy the citric acid cycle. Thus, amino acid synthesis is affected, resulting in brain edema and abnormal myelination.^[7]^ The acute phase is characterized by diffuse edema and locally severe edema.^[8,9]^

We present an extremely rare case of MSUD which involved the cervical cord, while most cases of nerve edema occur only in the brain. Different types of MSUD have different symptoms and different treatment methods, so early diagnosis is needed to reduce the neurological damage of abnormal metabolism. Diffusion-weighted imaging (DWI) has characteristic symmetric hyperintensity which is efficient to indicate the disease before it is confirmed by mass spectrometry and genetic testing.

## 2. Case report

The patient was an 8 days old female who with nasal congestion and poor response for 3 days. The child getting cold after a bath three days ago which leads nasal congestion, slightly shortness of breath, poor breast intake, poor mental reaction and hoarse crying. But chest CT was normal. The mother gave a natural birth at 40 + 1 weeks who was G1P1. The child had no birth asphyxia with a weight of 3.5 kg. The Apgar score was unknown and the child was mixed fed. The parents were healthy and denied family history of disease. The doctor examined the baby and found that the child has poor response, hoarse cry, shortness of breath (48 times/min), nasal congestion, scattered pustules on the whole body skin especially the face and neck. The children had poor mental response, poor lactation, and were easily awakened during the disease. Laboratory tests mainly included peripheral blood glucose, blood gas analysis, blood routine, liver and kidney function, and myocardial zymography. The peripheral blood glucose (Glu) was 1.7 mmol/L, PH 7.235, partial pressure of oxygen (PaO_2_) 123.3 mm Hg, partial pressure of carbon dioxide (PaCO_2_) 13.9 mm Hg, base excess (BE) -18.44 mmol/L, lactic acid (Lac) 2.0 mmol/L, white blood cell count (WBC) 1.40 × 10^10^/L, neutrophil percentage (N%) 10%, red blood cell count (RBC) 4.40 × 10^12^/L, hemoglobin (Hb) 155 g/L, platelet (PLT) 443 × 10^9^/L, total bilirubin (TBIL) 104.50 umol/L, direct bilirubin (DBIL) 7.50 umol/L, indirect bilirubin (IBIL) 97.00 umol/L, serum total protein (TP) 48.70 g/L, serum albumin (ALB) 34.60 g/L, uric acid (UA) 571.00 umol/L, myocardial enzyme spectrum - lactate dehydrogenase (LDH) 364.00 U/L, creatine kinase (CK) 240.00 U/L, creatine kinase isoenzyme (CK-MB) 29.99 U/L, α-hydroxybutyrate dehydrogenase (HBDH) 317.00 U/L. The results suggesting low blood sugar (LBS), metabolic acidosis combined with respiratory alkalosis, the whole blood three lines decreased, liver and kidney function and myocardial injury. She was kept warm, monitored by ECG and blood glucose, treated with acidosis, atomized by terbutaline and budesonide, regulated intestinal function with bifidobacterium triple viable bacteria, local skin cared with fusidic acid cream, fed with hyposensitive milk 60 mL Q3H through the mouth.

**Figure 1.**
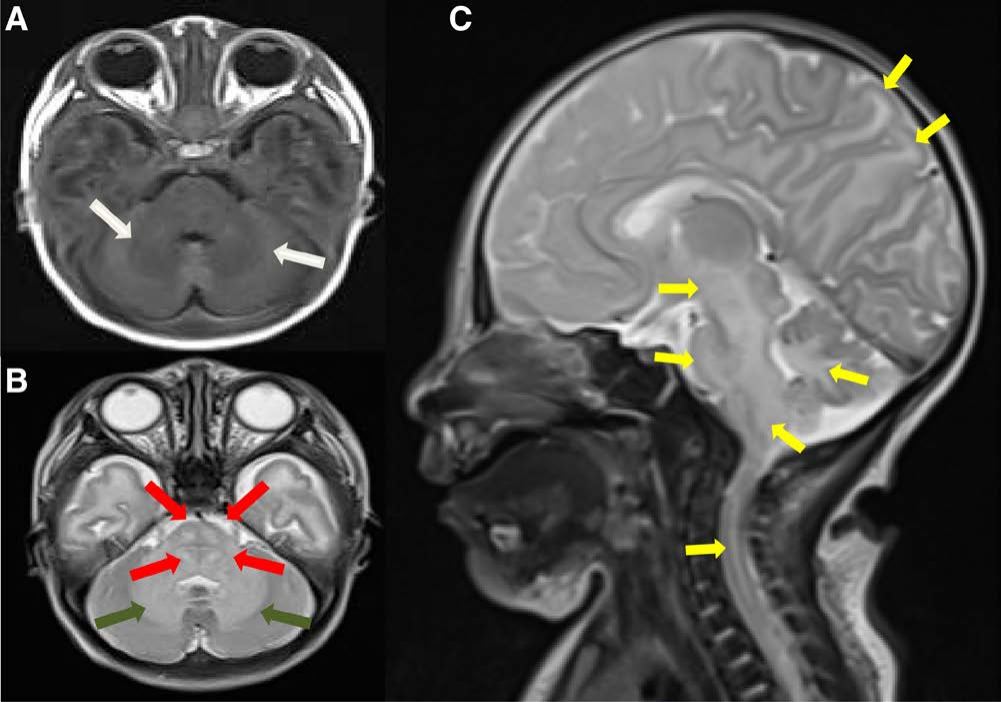
The brain T1WI and T2WI images of an 8-day-old female with MSUD. (a) The cerebellum and pons showed slightly symmetric low signal intensity (white arrow) on T1WI of cranial transection. (b) The corresponding parts of T2WI showed slightly high signal intensity (cyan arrow), and the pons showed symmetrical circular high signal intensity (red arrow), which looked like “button”. (c) The median sagittal MRI of the brain showed there were extensive patches and cords of T2 hyperintensity in the anterior and posterior central gyrus, cerebellar dentate nucleus, brainstem, and cervical spinal cord (yellow arrow). MSUD = maple syrup urine disease, MRI = magnetic resonance imaging, T1WI = T1 weighted imaging, T2WI = T2 weighted imaging.

**Figure 2.**
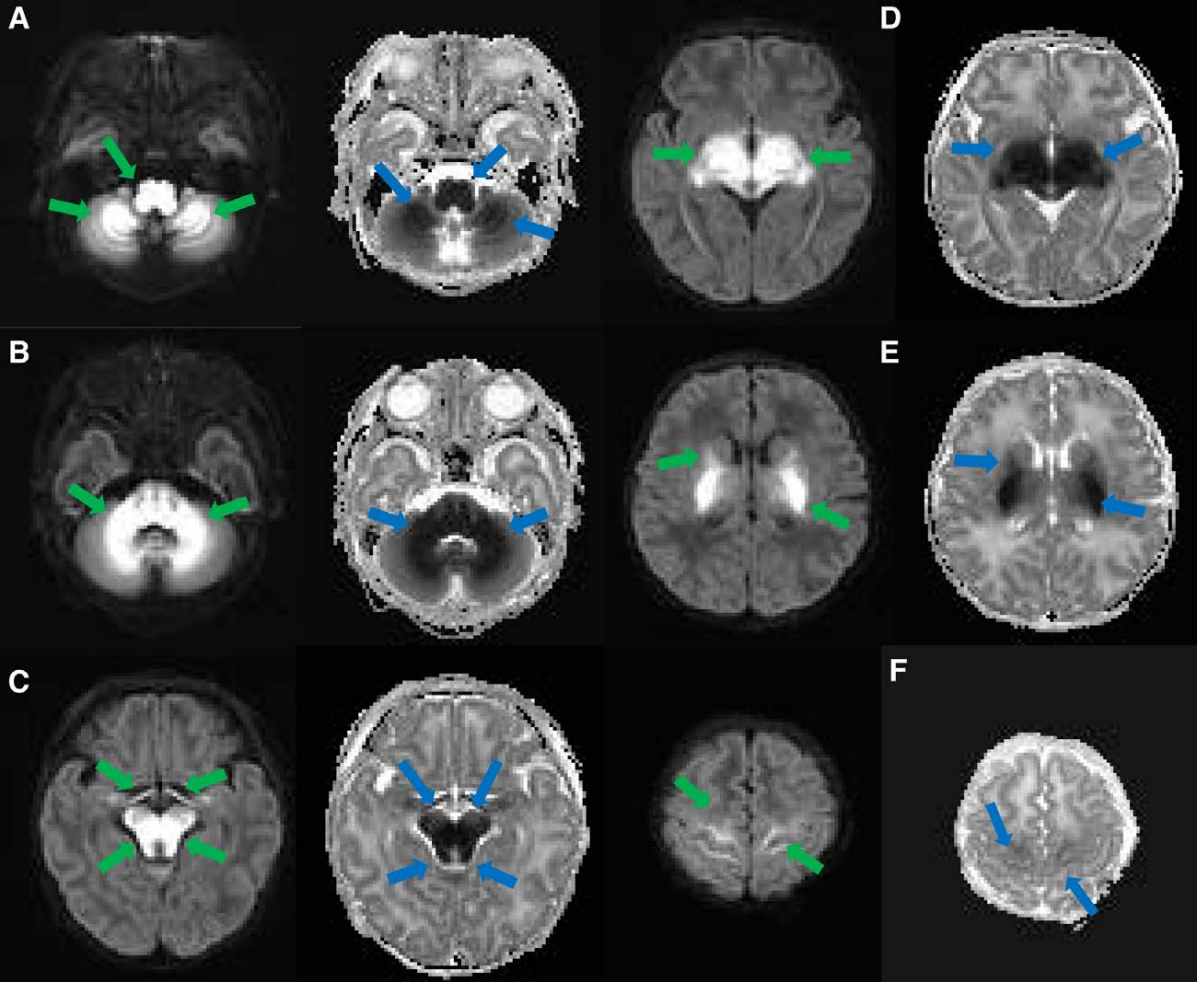
Axial DWI and ADC images of an 8-day-old female with MSUD. (a‐f) DWI and corresponding ADC images showing restricted diffusion in the brainstem, bilateral cerebellar hemispheres, basal ganglia, thalamus precentral gyrus and postcentral gyrus; DWI showed high signal intensity (green arrow), ADC showed low signal intensity (blue arrow), myelin or cytotoxic edema. ADC = apparent diffusion coefficient, DWI = diffusion-weighted imaging, MSUD = maple syrup urine disease.

She got worse the day after she was admitted. Her mental response was worse than before and her voluntary activity was less. Limbs shaking significantly after stimulation and was irritable. Brain MRI (Figs. 1 and 2) presented abnormal signal intensity mainly involving the brainstem, bilateral cerebellar hemispheres, basal ganglia, thalamus precentral gyrus and postcentral gyrus. T1 weighted imaging (T1WI) sequence showed symmetric patchy low signal intensity. T2 weighted imaging (T2WI) sequence showed signal intensity slightly increased. DWI sequence showed hyperintensity while the apparent diffusion coefficient (ADC) sequence showed the opposite. On the basis of the previous treatment plan, the doctor added ceftriaxone sodium anti-infection therapy and intravenous (IV) glucose energy support therapy, reduced the milk volume to 30 mL Q3H oral feeding for the patient. Nevertheless the baby refused to drink milk. On the basis of the original laboratory tests, doctors improved the detection of genetic metabolic diseases (GMD) by tandem mass spectrometry of blood and urine. At the same time the infants were fasted, the rotation speed of glucose pump was adjusted to maintain blood glucose and blood glucose Q8H was monitored. Mannitol was injected intravenously to reduce intracranial pressure, blood pressure Q8H was monitored, and lumbar puncture was performed. The cerebrospinal fluid (CSF) biochemistry, blood gas, bacterial smear, plasma procalcitonin (PCT) examinations were all normal. And the routine laboratory tests results showed no significant change from before.

The crying was high and discontinuous on 3rd day after admission, with groaning breathing and no active sucking reflex. Tandem mass spectrometry analysis of blood and urine showed that the disease was MSUD. The results showed that leucine (Leu) was 1841.80 umol/L and valine (Val) was 512.48 umol/L. In particular, 2-hydroxyisovaleric acid-2, 2-ketisovaleric acid-ox-2, 2-keto-3-methylvaleric acid-2, 2-ketisohexanoic acid-ox-2 increased significantly high. And the lactate-2, 3-hydroxybutyrate-2, 2-hydroxybutyrate-2 increased which suggest the ketonuria. The increase of 4-hydroxyphenyllactic acid and 4-hydroxyphenylpyruvate indicated liver injury. A few days later, the overall gene test results of genetic diseases showed that a homozygous copy number of BCKDHB gene was lost. Multiple heterozygous point mutations were found at the same time. And the mitochondrial DNA was normal. Doctor recommended the milk powder with the removal of BCAAs, regular measurement of serum BCAAs concentration and liver transplantation, if possible. The patient received a liver transplant one year later. Then she was in stable condition and had no restrictions on diet. However, the patient’s growth and development are slow. The timeline of diagnoses and interventions showed in Table 1.

**Table 1:** The timeline of diagnoses and interventions.

Time	Symptoms	Examination	Intervention
Be born	Natural delivery	-	Mixed fed
5th day	Getting cold	Chest CT: Normal	-
8th day	Scattered pustules, poor response and intake	Lab exam: LBS, metabolic acidosis, abnormal blood routine	Symptomatic treatments
9th day	Worse	Brain DWI-MRI: symmetrical high signal intensity CSF detection and plasma PCT: Normal	Anti-infection, IV Glu, fasted, IV mannitol
10th day	Worse	GMD Blood and urine MS/MS detection: MSUD	Milk without BCAA fed
17th day	Better	GMD gene detection: loss BCKDHB	-
Two years	Stable, growth slowly	-	Liver transplant 1 year before

BCKDHB = branched chain keto acid dehydrogenase E1 beta polypeptide, CT = computed tomography, CSF = cerebrospinal fluid, DWI-MRI = diffusion weighted imaging-magnetic resonance imaging, Glu = glucose, GMD = genetic metabolic diseases, IV = intravenous, LBS = low blood sugar, MS/MS = tandem mass spectrometry, MSUD = maple syrup urine disease, PCT = plasma procalcitonin.

## 3. Discussion

The brain MRI DWI findings of this patient with MSUD were consistent with the cytotoxicity or intramedullary edema reported in the previous literature rather than vascular or interstitial edema.^[2,10]^ The decrease in activity of Na+/K+ATPase causes an increase of the intracellular Na+ concentration. Diffuse brain edema and strong local edema occur with the liquid transfer to the cells, which also known as MSUD edema. There are four features of brain MRI in this disease. The T1WI and T2WI signal intensity are abnormal with bilateral symmetry slightly longer in the myelination process of normal newborn term infants. The corresponding parts appear in the deep white matter of cerebellum, central anterior posterior gyrus, brain stem, thalamus, internal capsule, globus pallidus, etc. The range of infratentorial lesions is relatively larger and the boundary is clear. Edema distributes along the nerve fiber bundle. It shows symmetrical round dot like hyperintensity on axial T2WI when involves bilateral pyramidal tracts, tegmental tracts and visual tracts which forming a typical “button” shape. The DWI and ADC show hyperintensity and hypointensity respectively. The DWI of brain edema is specific and symmetrical hyperintensity. It is caused by edema in the myelin sheath, limited diffusion of water molecules, and toxic intracellular edema. DWI is the optimal detection method for neonatal MSUD encephalopathy.^[11]^ Edema mainly involves the white matter of cerebellum, brain stem, globus pallidus, internal capsule and thalamus which usually occurs in myelinated areas of normal term neonates.^[12]^

Newborn MSUD is often a classic type with the most serious clinical symptoms. It is characterized by progressive seizures, high muscular tension, feeding difficulties and others, which would worse about 1 week after birth. They often become unconscious or die of brain edema within a few weeks if not actively treated. Most patients still suffer serious and permanent brain damage even control the symptoms.^[5]^ The traditional treatments are peritoneal dialysis machine hemodialysis and glucocorticoid treatment in the acute stage of the disease. While strict diet therapy is required in the chronic phase. The treatment plan is to feed milk powder without branched chain amino acids. The purpose is to maintain the plasma BCAA concentration within the range of no neurotoxicity, and provide sufficient nutrients. This plan should be combined with vitamin B1 and L-carnitine for long-term treatment at the same time.

In this case, multiple systems were involved, and the possibility of genetic metabolic diseases was gradually realized with the improvement of various examinations. DWI sequence is superior to T1WI and T2WI in displaying the scope of MSUD brain injury and its contrast with surrounding tissues. It can indicate the disease through the characteristic manifestations of MR but the differentiation from others must be paid to. The genetic disease overall genetic tests with blood and urine tandem mass spectrometry can confirm the diagnosis of MSUD. In addition, liver transplantation is an effective treatment for it. This treatment can prevent potential new brain injury and promote neural development.^[13–15]^ The patient received liver transplantation, making her physical condition improved. Nevertheless, the disease still causes growth retardation which needs to be addressed by more advanced and sophisticated treatment programs. The patient’s future condition needs further follow-up observation.

## Author contributions

**Investigation:** Fang Li.

**Validation:** Jidong Peng, Jingjing Zhou, Lianggeng Gong, Xiaoyu Liu.

**Writing – original draft:** Qiao Liu.

## References

[1] Strauss KA Puffenberger EG Morton DH. Maple syrup urine disease. Develop Med Child Neurol. 1959;1:104–5.

[2] Campanholi D Margutti A Silva WJ. Molecular basis of various forms of maple syrup urine disease in Chilean patients. Mol Genet Genomic Med. 2021;9:e1616. 33955723 10.1002/mgg3.1616 PMC8172190

[3] Strand JM Skinnes R Scheffler K. Genome instability in Maple Syrup Urine Disease correlates with impaired mitochondrial biogenesis. Metabol Clin Exp. 2014;63:1063–70. 10.1016/j.metabol.2014.05.003 24928662

[4] Vilela TC Scaini G Furlanetto CB. Apoptotic signaling pathways induced by acute administration of branched-chain amino acids in an animal model of maple syrup urine disease. Metab Brain Dis. 2017:1–8. 10.1007/s11011-016-9892-0 27510712

[5] Schönberger BSBS. Dysmyelination in the brain of adolescents and young adults with maple syrup urine disease. Mol Genet Metab. 2004;1:69–75. 10.1016/j.ymgme.2004.01.016 15110325

[6] Klee D Thimm E Wittsack HJ. Structural white matter changes in adolescents and young adults with maple syrup urine disease. J Inherit Metab Dis. 2013;6:945–53. 10.1007/s10545-012-9582-y 23355088

[7] Zinnanti WJ Jelena L Kathleen G. Dual mechanism of brain injury and novel treatment strategy in maple syrup urine disease. Brain. 2009;132(Pt 4):903–18. 19293241 10.1093/brain/awp024 PMC2668944

[8] Righini A Ramenghi LA Parini R. Water apparent diffusion coefficient and T2 changes in the acute stage of maple syrup urine disease: evidence of intramyelinic and vasogenic-interstitial edema. J Neuroimaging. 2010;13:162–5. 12722501

[9] Ha JS Kim TK Eun BL. Maple syrup urine disease encephalopathy: a follow-up study in the acute stage using diffusion-weighted MRI. Pediatr Radiol. 2004;34:163–6. 14504844 10.1007/s00247-003-1058-7

[10] Kilicarslan R Alkan A Demirkol D. Maple syrup urine disease: diffusion-weighted MRI findings during acute metabolic encephalopathic crisis. Jpn J Radiol. 2012;30:522–5. 22476847 10.1007/s11604-012-0079-2

[11] Xia W Yang W. Diffusion-weighted magnetic resonance imaging in a case of severe classic maple syrup urine disease. J Pediatric Endocrinol Metabolism. 2015;28. 10.1515/jpem-2014-0461 25879313

[12] Brismar J Aqeel A Brismar G. Maple syrup urine disease: findings on CT and MR scans of the brain in 10 infants. AJNR Am J Neuroradiol. 1990;11:1219–28. 2124065 PMC8332126

[13] Molema F Martinelli D Hrster F. Liver and/or kidney transplantation in amino and organic acid-related inborn errors of metabolism. An overview on European data. J Inherit Metab Dis. 2021;3:593–605. 10.1002/jimd.12318 PMC8247334 32996606

[14] Ewing CB Soltys KA Strauss KA. Metabolic control and “ideal” outcomes in liver transplantation for maple syrup urine disease. J Pediatr. 2021;237:59–64.e1. 34153280 10.1016/j.jpeds.2021.06.028 PMC9795541

[15] Medina MF Castro G Falcon F. Maple syrup urine disease: Characteristics of diagnosis and treatment in 45 patients in Chile. Am J Med Genet C Semin Med Genet. 2021;187:373–80. 34288399 10.1002/ajmg.c.31933

